# The McDowell Monument

**Published:** 1878-09

**Authors:** 


The McDowell Monument, to be erected at Danville, Ky., next spring, will consist of a shaft of granite, thirty feet high. The style will be Doric. On its base is the following inscription: "Beneath this shaft rests Ephraim McDowell, M.D., the father of ovariotomy, who, by originating a great surgical operation, became a benefactor of his race, known and honored throughout the civilized world." On the right side of the base is a laurel wreath, with the inscription: "A grateful profession reveres his memory and treasures his example." On the left side is an historic inscription, with the date of his birth, attendance at the University of Edinburgh, and his first ovariotomy in 1809. On the posterior face is the inscription: "Erected by the Kentucky Medical Society, A. D., 1879." An "ovarian jubilee," says the Detroit *Lancet*, is expected at the next annual meeting of the Society.—*American Medical Bi-Weekly.*

